# Biointegrative Fixation for Tibial Tubercle Osteotomy Is Effective and May Lower Removal Rate

**DOI:** 10.1016/j.asmr.2025.101267

**Published:** 2025-09-24

**Authors:** Scott M. Feeley, Rehan Dawood, Parth Sharma, Jamie M. Mwendwa, Christopher M. Kuenze, Edward S. Chang, Brandon J. Bryant

**Affiliations:** a Department of Orthopaedic Surgery, Walter Reed National Military Medical Center, Bethesda, Maryland, U.S.A.; b Inova Sports Medicine, Fairfax, Virginia, U.S.A.; c Villanova University, Villanova, Pennsylvania, U.S.A.; d Department of Kinesiology, University of Virginia, Charlottesville, Virginia, U.S.A.; e School of Medicine, University of Virginia, Inova Campus, Falls Church, Virginia, U.S.A.

## Abstract

**Purpose:**

To report the safety and efficacy of biointegrative fiber-reinforced implants for tibial tubercle osteotomy (TTO) and perform a cost-benefit analysis.

**Methods:**

Patients treated with TTO for all indications by a single surgeon from May 2017 to July 2024 were retrospectively reviewed. There were no exclusion criteria. In 2023, the surgeon switched TTO fixation from two 4.5-mm metal compression screws to 2 biointegrative, partially threaded 4.0-mm headless compression screws, made of continuous mineral fibers comprised of elements found in natural bone (SiO_2_, Na_2_O, CaO, MgO, B_2_O_3_, and P_2_O_5_), and bound together by PLDLA [poly (L-lactide-co-D,L-lactide)] (70:30 L:DL) in 50% weight by weight ratio. Postoperative protocols were consistent across fixation types, with full weight-bearing as tolerated in full extension for 6 weeks and range of motion from 0 to 90° allowed immediately. Patients were followed longitudinally for osseous union, recurrent instability, and return to the operating room.

**Results:**

Sixty-two TTOs were analyzed (44 metal, 18 biointegrative); 61.3% of patients were female, and the median age was 23.3 years (interquartile range 22.5). One patient with biointegrative implants underwent irrigation and debridement with implant retention at 9 days postoperatively. Mean follow-up for biointegrative fixation was 1.3 ± 0.4 years (range: 0.5-1.8) and for metal fixation was 3.5 ± 1.8 years (range: 0.4-6.7), *P* < .001. All patients achieved clinical union by 6 months without differences in time to clinical union (*P* = .159). Hardware removal rates differed between metal versus biointegrative groups (29.5 vs 0%, *P* = .009) but otherwise did not differ for rates of superficial infection (2.3 vs 0%, *P* = .519) or deep infection (2.3 vs 5.6%, *P* = .507).

**Conclusions:**

Biointegrative screws are a safe and effective alternative to metal screws for TTO in short-term follow-up. The use of biointegrative fixation may reduce the need for secondary hardware removal procedures commonly reported with 4.5-mm metal screws, albeit at a potentially increased cost to the health system.

**Level of Evidence:**

Level III, retrospective comparative study.

Tibial tubercle osteotomy (TTO) is a well-described surgical treatment option for a broad range of patellofemoral joint disorders.^1^^,^^2^ In clinical practice, the osteotomy is most commonly fixated using 2 or 3 metal compression screws (CS), providing the required fixation strength.^3^ When 2 screws are used, they are often larger screws that have been associated with symptomatic hardware.^4^ Multiple potential complications can follow metal TTO fixation, including superficial wound infections, skin irritation and necrosis, symptomatic hardware, tibial or tuberosity fractures, nonunion, neurovascular complications, and deep infection, all of which can result in the need for revision procedures and hardware removal.^5^^,^^6^

Prominent and symptomatic hardware often results in the need for a secondary removal of hardware (ROH).^4^ Bioabsorbable implants purportedly limit the prominence of hardware as they degrade with time. In fixation for TTO, the use of bioabsorbable screws has previously been evaluated biomechanically and showed adequate fixation strength for early mobilization.^3^ Further, they have been evaluated clinically in fixation of pediatric tibial tubercle fractures and were found to eliminate the need for hardware removal compared with metal screws.^7^ However, bioabsorbable implants for other indications are not without their own distinct complication profile.^8^ In the setting of anterior cruciate ligament reconstructions, approximately 10% of patients with bioabsorbable interference screws were found to develop a screw-related problem.^9^ Related problems included implant migration, cyst accumulation, and peri-implant osteolysis, which can occur as a result of an acidic microenvironment and foreign body reaction.^10^

Biointegrative mineral fiber-reinforced implants may limit some of these adverse tissue responses^11^^,^^12^ because they are thought to undergo a more gradual biointegration without adverse inflammatory response. Recent evidence suggests that more recent bioabsorbable implants may have lower complication rates than historical alternatives.^13^ However, the use of biointegrative mineral fiber-reinforced implants has not been reported for fixation in TTO, and there has been limited reporting on the cost-benefit of such implants. Therefore, the purpose of this study was to report the safety and efficacy of biointegrative fiber-reinforced implants compared with metal screws for TTO and perform a cost-benefit analysis. Our hypothesis was that union rates and outcomes would be similar between fixation types while reducing the need for hardware removal with biointegrative screws, and we projected that the use of biointegrative screws would not increase costs to the health system compared with metal CS because of a decrease in hardware removal rate with biointegrative screws.

## Methods

Institutional review board approval was obtained for this retrospective comparative study WCG IRB #U21-10-4593). Patients were included if treated with TTO (Current Procedure Terminology [CPT] code 27418) by the senior surgeon (B.B.) at a private, academic model institution from May 2017 to July 2024. No patients were excluded in an effort to capture the entire complication profile postoperatively for this procedure. The senior surgeon is fellowship-trained in sports medicine with more than 17 years in practice. In 2023, the surgeon switched TTO fixation from 2 metal screws to 2 biointegrative screws.

Patient sex, age, BMI, smoking status, and presence of diabetes were recorded. The tibial tubercle-trochlear groove distance was recorded on preoperative advanced imaging. The indication for surgery was grouped into patellar instability, chondral offloading, or both. Concomitant procedures were recorded.

Postoperatively, patients were followed longitudinally with standard postoperative visits at 2 weeks, 6 weeks, 12 weeks, and 6 months. Radiographs were typically obtained at the 6- and 12-week postoperative visits. Clinical or radiographic union was verified. All patients with biointegrative implants were followed until radiographic union. Clinical union was defined as lack of tenderness to palpation about the osteotomy site. Radiographic union was defined as bridging bone across the osteotomy site on radiographs. Radiographs were evaluated for union by an orthopaedic surgery sports medicine fellow (R.D.). The presence of recurrent patellar instability was evaluated for at all postoperative visits and the time to recurrence was recorded if present. Superficial infection requiring only antibiotics was distinguished from deep infection requiring return to the operating room for irrigation and debridement with or without removal of hardware. All cases of reoperation and their timing were recorded and grouped by irrigation and debridement, revision procedures, or ROH (CPT code 20680). These data were collected retrospectively in a single effort immediately before statistical analysis. Finally, the cost of implants at the study health system were obtained in addition to the median cost for ROH and TTO procedures by CPT code for the year 2024 in both the ambulatory surgical center and hospital facility settings.

### Surgical Technique

**Fig 1:**
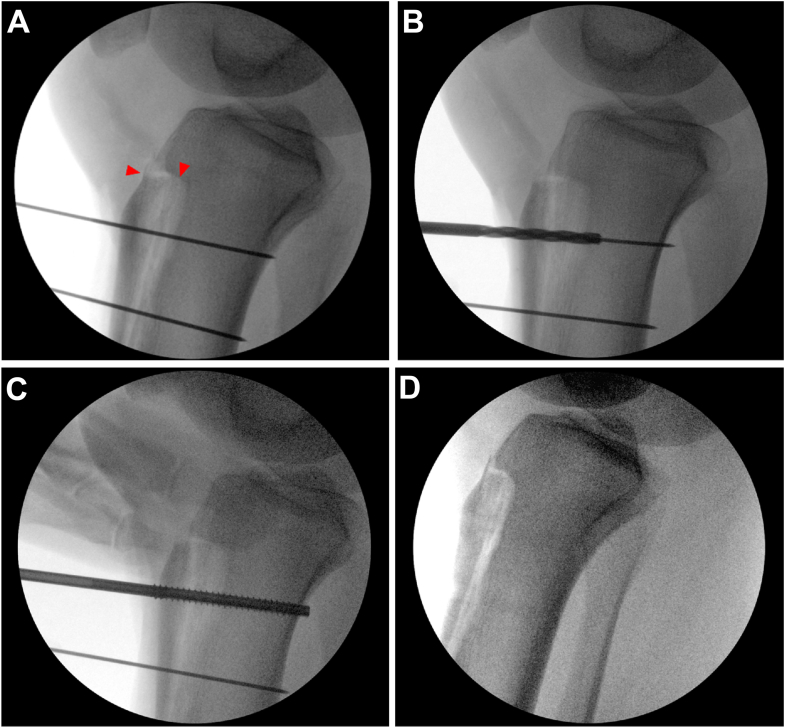
After tibial tubercle osteotomy (red arrowheads), anteromedialization was held in place with 2 Kirschner wires (A) for cannulated drilling (B) as shown on lateral fluoroscopy of the knee with the patient in the supine position. Tapping of each screw tract was performed (C) for biointegrative compressive screw placement for fixation. After placement, 2 radiolucent tracks can be seen at the site of the inferior and superior biointegrative screws (D).

The patient was placed supine on a standard operating room table. Diagnostic arthroscopy was performed before TTO and intraarticular pathology was treated at that time. For TTO, an 8-cm longitudinal incision was made centered over the tibial tubercle to identify and free the medial and lateral borders of the patellar tendon. The anterior compartment was elevated from the lateral tibia using a Cobb elevator to expose the osteotomy site. A freehand cut at 10-15° was made from medial to lateral across the tibial. The periosteum was left intact at the distal osteotomy and straight osteotomes were used to complete the superomedial and superolateral portions of the osteotomy before completing the distal aspect of the osteotomy. The tibial tubercle was medialized approximately 9 mm, then secured with 2 Kirschner wires at the desired screw location and trajectory. The superior screw path was drilled, then length was measured, and finally tapping and countersinking were performed before metal or bio-integrative screw placement (Fig 1). This process was repeated for the inferior screw and verified under fluoroscopy. For the biointegrative screws, any remaining screw prominence was able to be removed with rongeur until flush with the tibia tubercle. No bone grafting was performed at the osteotomy site.

In 2023, the surgeon switched TTO fixation from using 2 fully threaded, headed 4.5-mm metal CS without washers (Synthes, Warsaw, IN) to using 2 biointegrative, partially threaded 4.0-mm headless CS (Ossio, Caesarea, Israel). These biointegrative implants are made of continuous mineral fibers composed of elements found in natural bone (SiO_2_, Na_2_O, CaO, MgO, B_2_O_3_, and P_2_O_5_), and bound together by PLDLA [poly (L-lactide-co-D,L-lactide)] (70:30 L:DL) in 50% weight-by-weight ratio.

#### Postoperative Protocol

Postoperative protocols were consistent across fixation type. Patients were full weight-bearing as tolerated in full extension for 6 weeks, with range of motion from 0 to 90° allowed immediately. At 6-week follow-up, bracing was discontinued and activity advanced with evidence of clinical evidence of patellar stability and evidence of progression toward osseous union. Return to running was allowed beginning at 12 weeks.

#### Statistical Analysis

Descriptive statistics were generated for the entire cohort and by implant group. Comparisons of continuous data were conducted with t-tests or Mann-Whitney *U* test for non-normally distributed variables and categorical data with χ^2^ or Fisher exact tests. Kaplan-Meier survival plots were created by implant type for time to ROH. Multivariate logistic regression analysis was performed to identify factors associated with ROH using demographics and implant group. Qualitative information related to the postoperative course was analyzed for any complication or reoperation for patients with both implant types for fixation. A cost-benefit analysis was performed to compare the cost to the health system for each method of TTO fixation, taking into account implant costs, the median cost of TTO and ROH by surgical setting, and the removal of hardware rate by implant group. A-priori alpha level was established as *P* < .05. Statistical analyses were performed using jamovi v2.2.5.0.

## Results

**Fig 2:**
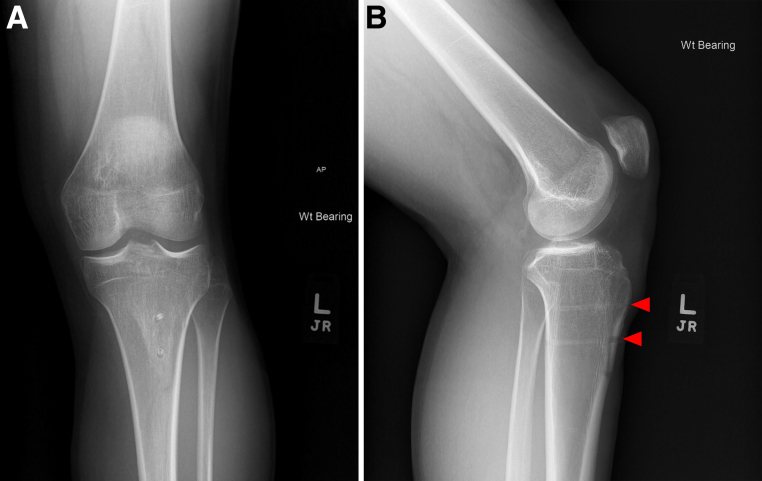
Representative (A) anteroposterior and (B) lateral postoperative radiographs of tibial tubercle osteotomy fixation showing osseous union with an osteotomy line and shadow of 2 radiolucent biointegrative screws (red arrowheads) at 4.6 months.

Sixty-two TTOs were analyzed (44 metal, 18 biointegrative). There were no exclusions and all were primary TTO. The overall cohort was 61.3% female, with median age of 23.3 years (interquartile range [IQR] 22.5 years) and BMI 25.7 (IQR 5.0). Because of limited numbers in each subgroup, further analysis remained aggregated by sex. Only 1 patient (1.6%) was an active smoker, and no patients had diabetes. Mean follow-up duration was 2.9 ± 1.8 years. Mean preoperative tibial tubercle-trochlear groove was 18.1 ± 3.1 mm. Patients were indicated for TTO for chondral offloading (11.3%), isolated patellar instability (22.6%), or both (66.1%). Concomitant procedures included 22 medial patellofemoral ligament reconstructions, 9 lateral retinacular releases or lengthening, 3 osteochondral allograft transplantation surgery, 5 debridements, 3 chondroplasties, 3 loose body removals, and 1 meniscus root repair. When we compared implant groups, only follow-up duration (*P* < .001) and indications for surgery (*P* = .013) were significantly different between groups (Table 1). All patients achieved clinical union and clearance for return to activity without restriction by 6 months without differences in time to clinical union between groups (*P =* .159), as shown in Table 2. All biointegrative TTOs achieved radiographic union (Fig 2).

**Table 1:** Comparison of TTO Fixation Implant Groups

Characteristic	Metal	Biointegrative	*P* Value
Age, y (IQR)	22.4 (20.1)	2.59 (23.5)	.443
BMI (IQR)	25.8 (6.1)	23.9 (4.4)	.092
TT-TG, mm	17.8 ± 2.9	18.9 ± 3.6	.195
Follow-up, yr (range)	3.5 ± 1.8 (0.4-6.7)	1.3 ± 0.4 (0.5-1.8)	<.001∗
Sex: % female	61.4%	61.1%	.985
Indication for surgery, n			
Chondral offloading	7	0	.013∗
Patellar instability	6	8	
Both	31	10	
ROH, %	29.5%	0.0%	.009∗
Recurrent instability, %	13.6%	0.0%	.099
I&D, %	2.3%	5.6%	.507
Revision, %	4.5%	0.0%	.358

I&D, incision and drainage; IQR, interquartile range; ROH, removal of hardware; TTO, tibial tubercle osteotomy.

∗ Denotes statistically significant values.

**Table 2:** Comparison of Time to Clinical Union by Fixation Implant Groups

Time to Clinical Union	Metal	Biointegrative
3 mo	1 (2.3%)	0 (0%)
4 mo	4 (9.1%)	3 (16.7%)
5 mo	0 (0%)	2 (11.1%)
6 mo	39 (88.6%)	13 (72.2%)

**Fig 3:**
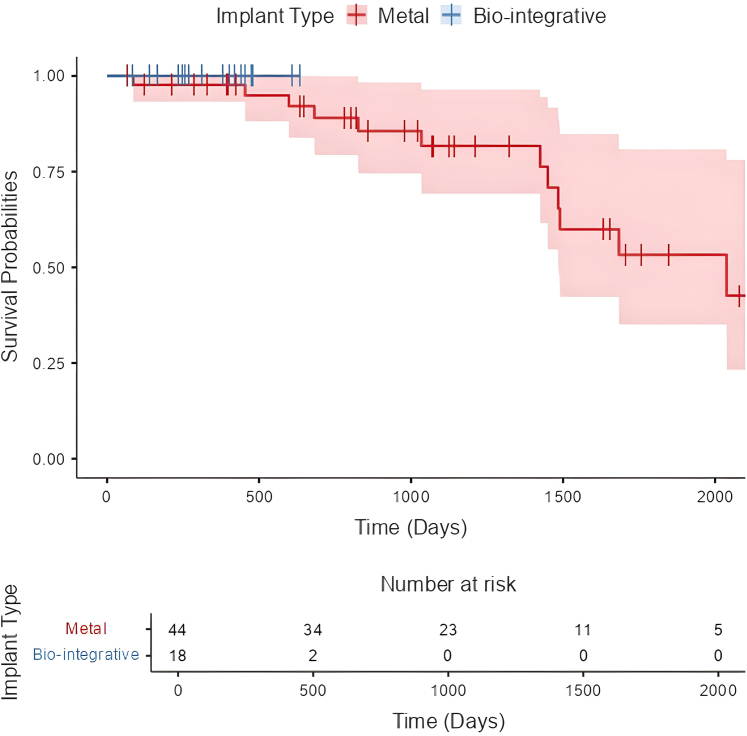
Kaplan-Meier plot showing survivorship by implant group over time.

Six patients (9.7%) had recurrent instability at a mean 732 ± 487 days, all of whom had metal implants for TTO fixation. ROH was required for 13 of 44 (29.5%) metal fixations at a mean 505 ± 358 days postoperatively (range 210-1561 days) (Fig 3). Comparatively, there were no removals of biointegrative implants at time of reporting (*P* = .009). Of the 13 ROH, 1 was performed in the hospital setting for a ROH with conversion to total knee arthroplasty and 12 were performed in the ambulatory surgical center with same day discharge. Two patients with metal fixation (3.2%) required a revision procedure at a mean 830 ± 1,034 days. One had medial patellofemoral reconstruction performed for the revision for recurrent instability, and the second was the previously mentioned conversion to total knee arthroplasty. There were no manipulations under anesthesia or lysis of adhesions performed. With the numbers available, a multivariate logistic regression analysis was underpowered to identify any specific risk factors associated with increased risk of ROH.

There were no instances of biointegrative screw breakage with tightening. One metal and no biointegrative fixations (2.3 vs 0%, *P* = .519) required antibiotics for a superficial infection. One metal and one biointegrative fixation (2.3 vs 5.6%, *P* = .507) had concern for a deep infection requiring irrigation and debridement in the operating room. The 1 patient with biointegrative implants who underwent irrigation and debridement did so with implant retention at 9 days postoperatively. She had a fever at home with increase in knee pain and a C-reactive protein of 1.2 mg/dL. She underwent arthroscopic knee irrigation and tibial incision open irrigation and debridement. Intraoperatively, there was no sign of synovitis or intra-articular infection and separately there was no visualized fluid collection or purulent drainage around the osteotomy site. She was admitted overnight for intravenous vancomycin and discharged on 2 weeks of trimethoprim-sulfamethoxazole. One intraoperative culture from the osteotomy site had light growth of *Staphylococcus epidermidis*, although the patient required no additional treatment and had hiked 14 miles in a 2-day span at final follow-up at 7 months.

A Synthes 4.5-mm stainless steel cortical screw is $19 for a total of $38 in implant costs for TTO with metal fixation. An Ossio 4.0-mm biointegrative screw costs $803 per implant for a total of $1,606 in implant costs for TTO with biointegrative fixation. At the study institution, the median cost to perform a TTO in the hospital setting is $19,337.36 and at an ambulatory surgical center is $11,165.47. The median cost to perform a ROH in the hospital is $5,434.41 and at an ambulatory surgical center is $3,628.11. Assuming all metal TTOs are performed in the ambulatory setting with a ROH rate of 29.53% with a ratio of 12:1 ROH being performed in the ambulatory/hospital setting as in this study, the average cost to the system for each TTO is $12,316.46. Similarly, assuming all TTOs are performed in the ambulatory setting with a ROH rate of 0% as in this study, the average cost to the system for each biointegrative TTO is $12,771.47. The difference in cost per procedure was $455.01 more for biointegrative TTOs.

## Discussion

The most important finding of this study was that biointegrative fixation for TTO was a viable alternative to metal screw fixation with similar union rates and without the need for removal of hardware. Biointegrative screws were effective at achieving osseous union and clearance for return to activity without restrictions by 6 months in all cases. However, although the use of biointegrative screws avoided the risk of secondary hardware removal at short-term follow-up, biointegrative screws were used at an increased cost to the health system.

Symptomatic hardware after TTO is a common issue often necessitating removal of hardware, which occurred at a rate of 29.5% of metal fixations in the present study. In a systematic review of TTOs, 10 studies reporting symptomatic removal of hardware pooled for an overall incidence of 36.7%.^6^ Rates of removal were significantly greater in cases in which anteriorization was performed compared with direct medialization. In an additional 2 studies of that review, all hardware were routinely removed by the surgeons.^14^^,^^15^ With the predominantly medialized transfer of the tubercle in the present study, this suggests that our reported hardware removal rates may increase with a technique prioritizing anteriorization.

In their study of 153 TTOs evaluating for risk factors for complications, Johnson et al.^4^ reported that 46% of patients experienced at least 1 complication after TTO. Of the entire cohort, 21% experienced painful hardware all of which had removal performed. However, after subgroup analysis by screw size, the rate of hardware removal increased to 27% with the use of two 4.5-mm screws and was significantly greater compared with the 2.6% rate of hardware removal with fixation with three 3.5-mm screws. This rate is comparable with the 29.5% rate of removal of 4.5-mm screws in the present study. In another study evaluating symptomatic hardware removal rates after TTO, Lehane et al.^16^ reported a significant reduction in removal associated with the use of headless screws (13.2% vs 1.7%). In the present study, metal fixation was with headed 4.5-mm screws which were countersunk without a washer. With a 0% removal rate in the 4.0-mm biointegrative group in the current study, some of the difference in rate of hardware removal may be attributable to a smaller screw diameter in addition to the biointegrative and headless nature of the implants used.

The timing of removal of hardware is an important consideration when evaluating the results in the present study given the short-term follow-up for the biointegrative implant group. With metal screws, the earliest hardware removal was performed at seven months and the latest at 4.3 years postoperatively. Nearly one-third of metal hardware removals in the present study occurred before one year follow-up. In a study with minimum 15-year follow-up for metal screws with TTO, ROH was performed in 59%.^17^ This rate is much greater than that reported in systematic reviews, which may indicate that rates of removal are impacted by duration of follow-up for metal screws. However, previous published studies have examined the timing of bio-integration for the mineral fiber-reinforced material used for the implant in the present study. In a preclinical study assessing a 2.1-mm thick, mineral fiber-reinforced plate implanted into sheep tibia, plate bioabsorption was found to peak between 52-78 weeks, the plate was no longer visible on microCT at 78 weeks, and only 10% to 20% remained on histological examination at 78 weeks with complete remodeling by 104 weeks.^11^ More importantly, there were no adverse tissue responses. In the setting of proximal interphalangeal joint arthrodesis, Štalc et al.^12^ found that implant biointegration improved between postoperative years 1 and 2 without any signs of adverse reaction. These data collectively suggest that any symptoms related to prominence of the biointegrative screw in the present study may be limited to a short-term duration before complete biointegration, although this will need to be fully evaluated with future study.

Infection after TTO is exceedingly rare, although it is an important consideration with bio-integrative implants which may be more difficult to remove depending on the degree of integration at time of infection. In a systematic review of 787 TTOs, only 8 (1.0%) were reported to have a postoperative infection and only one (0.1%) required surgery to address the infection.^6^ In the present study, 3 patients (4.8%) had a postoperative infection, one of which was in the biointegrative group and had irrigation and debridement performed with implant retention. Although that patient had no further adverse effects following treatment, the growth of *S epidermidis* on a single culture is of consequence for further study with the use of biointegrative implants.

Finally, when selecting implants for any procedure, costs must be appreciated in the context of outcomes. Orthopaedic surgeons play a critical role in cost containment through their ability to reduce implant-related health care costs.^18^^,^^19^ Several studies have shown that orthopaedic surgeons taking an active role in implant use and stewardship led to significant cost savings and price reductions for their institutions.^20^, ^21^, ^22^, ^23^ Although the present study did show a marked improvement in the rate of hardware removal in the biointegrative implant group, the increased costs of the implants were not recouped by the decrease in cost of secondary removal procedures.

### Limitations

There are several notable limitations to our study. The retrospective design is limited by inherent biases and relies upon the accuracy and completeness of clinical documentation. The most notable limitation is the difference in sample size and follow-up duration between implant groups, which may have skewed the complication profile toward the metal group, with its longer duration follow-up. Although there were no differences between groups for surgical-site infection or patellar instability, this lack of difference should be interpreted with caution in light of the significantly different follow-up durations between groups. In addition, without a single ROH in the biointegrative group, we were underpowered to perform multivariate analysis of risk factors for ROH to account for the potential confounder of follow-up duration. The cohort in the present study was fairly young and healthy with biointegrative fixation performed in the elective setting. Our results may not be externally valid to trauma populations or those with access to care concerns. Separately, TTO for all indications were included in analysis that may have had an unaccounted for impact on the results. However, with all TTOs having been performed by a single surgeon with similar postoperative protocols, it is unlikely that any differences between implant groups derived from these factors. Finally, the cost-benefit analysis relied on cost assumptions at one health system and may not be generalizable to other systems. The cost-benefit analysis in the present study was performed from the health system perspective and may differ if performed from the patient perspective, taking into account the additional costs to the patient from insurance co-pays and deductibles for additional imaging, pre- and postoperative appointments, a second surgery, and time off from an occupation. It also does not account for other advantages of biointegrative hardware, which include the lack of metal artifact scatter on advanced imaging.

## Conclusions

Biointegrative screws are a safe and effective alternative to metal screws for TTO in short-term follow-up. The use of biointegrative fixation may lower the need for secondary hardware removal procedures commonly reported with 4.5-mm metal screws, albeit at a potentially increased cost to the health system.

## Disclosures

The authors declare the following financial interests/personal relationships which may be considered as potential competing interests: S.M.F. received educational support from Supreme Orthopedic Systems LLC. E.C. reports a relationship with Avanos Medical Inc that includes consulting or advisory and received educational and research support from Arthrex. B.B. reports a relationship with OSSIO Inc that includes consulting or advisory, speaking and lecture fees, and travel reimbursement. B.B. also reports a relationship with Zimmer Biomet Holdings Inc that includes consulting or advisory and equity or stocks. All other authors (R.D., P.S., J.M.M., C.M.K.) declare that they have no known competing financial interests or personal relationships that could have appeared to influence the work reported in this paper.
